# Editorial: The impact of lifestyle changes on non-communicable diseases

**DOI:** 10.3389/fnut.2024.1471019

**Published:** 2024-08-28

**Authors:** Annalisa Noce, Giulia Marrone, Attilio Parisi

**Affiliations:** ^1^Department of Systems Medicine, University of Rome Tor Vergata, Rome, Italy; ^2^UOSD Nephrology and Dialysis, Policlinico Tor Vergata, Rome, Italy; ^3^Department of Exercise, Human and Health Sciences, Foro Italico University of Rome, Rome, Italy

**Keywords:** lifestyle changes, nutritional habits, physical activity, chronic non-degenerative diseases, obesity, muscle mass

Non-communicable diseases (NCDs) are a series of pathological conditions, mainly caused by unhealthy behaviors but they can also depend on genetic factors, sex, age and air pollution. Currently, NCDs are the leading cause of disability and death worldwide. Moreover, NCDs imply a significant expense for their treatment and care by the National Health Systems. Lifestyle is among the modifiable factors in determining the NCDs onset. Therefore, it is very important to implement campaigns aimed at raising the general population awareness about how a healthy lifestyle is essential to maintain a good state of health and to reduce NCDs incidence. Consequently, lifestyle changes represent a new challenge involving the scientific community. At this regard, an unhealthy diet and a sedentary lifestyle can be important contributors to the development of many diseases. On the contrary, a regular physical activity, a correct diet, a nutritional supplementation and a proper sleep duration and quality can act positively in preventing and/or counteracting NCDs.

Our Research Topic has collected a number of interesting publications highlighting how lifestyle modifications can be effective in counteracting the widespread of NCDs.

Several researchers have studied the association between the intake of certain foods with the occurrence of some NCDs. Due to the development of the social economy and improvement in the living standards, the eating habits of modern people have undergone tremendous changes compared to the past. Because of their job, Chinese physicians, are more prone to follow an unregulated lifestyle, including unhealthy eating habits and for this reason they have a high risk of developing these diseases. Chen et al. investigated the association between physicians' eating habits and their health and disease perception. According to the results, the prevalence of unhealthy eating habits was high, i.e., frequent eating out-of-home, irregular meals and eating too fast were significantly related to perceived sub-optimal health status and disease occurrence.

The study conducted by Wang et al. investigated the relationship between dietary differences and the risk of oral cancer. They extracted, from the UK Biobank database, 21 dietary exposures, including 10 dietary patterns, six vitamins, and five micronutrients. According to the results of the 10 analyzed dietary patterns, eight of them showed no significant association with the risk of developing oral cancer. Consumption of dark chocolate and sweet pepper exhibited an inverse relationship with oral cancer risk.

Moreover, degenerative diseases such as osteoarthritis can be influenced by the diet. Xie and Qin investigated the effects of 45 common dietary intake habits on six osteoarthritis-related phenotypes and they identified 59 potential causal associations. Results showed that muesli intake was negatively associated with knee osteoarthritis, spine osteoarthritis and total knee replacement, while, dried fruit intake reported a negative association with osteoarthritis of knee and total knee replacement.

According to Yang Y. et al., dietary factors, including intake of alcohol, non-oily fish, beef, fresh fruit, oily fish, salad/raw vegetables, dried fruit etc., contribute to determining or reducing the possibility of developing the various forms of hernia. Results evidenced that alcoholic drinks *per* week reduce the risk of inguinal hernia, while alcohol intake frequency does not affect the risk of the inguinal hernia. Cheese and dried fruit intake decrease the risk of ventral hernia, while cooked vegetable intake increase the risk of ventral hernia. Lastly, the intake of non-oily fish increases the risk of inguinal hernia.

Diet during pregnancy can also influence the occurrence of NCDs in unborn children. The study by Miyake et al. investigated the impact of maternal fiber intake during pregnancy on fetal neurodevelopment in Japanese population, showing that pregnant Japanese women had a lower fiber intake compared to the recommended one. Therefore, these data confirm how it is essential a correct diet during pregnancy in order to reduce the onset of health problems in newborns and how it is essential a multidisciplinary approach, which also includes the professional figure of the nutritionist during pregnancy.

Furthermore, interventions through oral supplementation or through a changing in the percentage of macronutrients in the diet seem to have a positive effect on several diseases. According to the study of Massimino et al., supplementation for 3 months with a low dose of medium-chain triglycerides (MCTs) has increased muscle mass and function in frail older adults. These findings indicate the potential practical use of MCTs in daily life for the treatment of sarcopenia. In fact, dietary lipid manipulation in the nutritional management of glycogen storage disease type III (GSD III) has been shown to be effective in reducing associated muscle damages. A low-CHO (32%)/high-fat (45%)/high-protein (23%) diet was safe, sustainable and effective in reducing muscle damages without worsening cardiometabolic profile in GSD III.

Similarly, physical activity plays a pivotal role in preventing or treating several NCDs, supporting the dietary treatment. In example, a low-calorie diet, which is commonly used in the treatment of obesity, induces a lean body mass loss, when it is not combined with an adapted exercise training. As evidenced by Monsalves-Álvarez et al., a 3 months high intensity interval training (HIIT) prevents a muscle mass loss caused by a hypocaloric-Mediterranean diet in overweight and obese women.

Recent findings evidence a relationship between lifestyle and the microorganisms diversity populating the intestine. Mancini et al., investigated the role of lifestyle (active vs. sedentary) on saliva microbiota composition in Italian school children. One-hundred-fourteen children were enrolled in a Turin neighborhood school. The results showed that children with an active lifestyle compared to sedentary children had an enrichment of the saliva microbiota species and genera mainly associated with a healthier profile, while the species, developed in the sedentary group, could be linked to human diseases.

Dietary habits, as well as the sleep quality, may increase the incidence of NCDs. The study conducted by Yang W. et al. has investigated the association between the sleep duration, the regularity of breakfast and overweight in 1,178 university students. The authors find out that only 34.1% of the study population ate a breakfast every day, while students that consumed breakfast from 1 to 3 times/week showed a higher risk to develop overweight. Moreover, short sleep duration may be the main reason for irregular breakfast, leading to overweight.

In conclusion, it is very important to follow a correct lifestyle not only to counteract the NCDs onset but also to act as an adjuvant therapy in the clinical management of NCDs patients.

## Author contributions

AN: Writing – original draft, Writing – review & editing. GM: Writing – original draft, Writing – review & editing. AP: Writing – original draft, Writing – review & editing.

